# A meta-analysis of normal human blood cholinesterase activities determined by a modified electrometric method

**DOI:** 10.25122/jml-2022-0215

**Published:** 2023-01

**Authors:** Hishyar Mohammed Salih Garmavy, Ammar Ahmed Mohammed, Hussein Mohammed Rashid, Fouad Kasim Mohammad

**Affiliations:** 1 Department of Pharmacology, College of Pharmacy, University of Duhok, Duhok, Iraq; 2 Department of Physiology, Biochemistry and Pharmacology, College of Veterinary Medicine, University of Mosul, Mosul, Iraq

**Keywords:** acetylcholinesterase, pesticides, organophosphates, carbamates, biomonitoring, meta-analysis

## Abstract

Measurements of blood cholinesterase (ChE) activities, including those of erythrocytes (EChE), plasma or serum (PChE), and whole blood (WBChE), are used to assess exposure to ChE-inhibiting pesticides. The purpose of this review was to report normal reference ChE activities in the blood of healthy adult humans, as determined by a modified electrometric method. We performed a systematic review following PRISMA guidelines. Single-group meta-analysis of means of PChE, EChE, and WBChE activities of adult healthy subjects was conducted using the random effects model. The programs used were Open-Meta Analyst and Meta-Essentials Version 1.5. Studies selected for analysis comprised 21, 19, and 4 reports of normal reference/baseline PChE, EChE, and WBChE activities in 690, 635, and 121 healthy adult males and/or females, respectively. The meta-analysis showed normal reference values of the means (effect sizes) with 95% CI of PChE, EChE, and WBChE activities of healthy adult subjects, which were 1.078 (1.015, 1.142), 1.075 (1.024, 1.125) and 1.331 (1.226, 1.436), respectively. By the subgroup analysis, heterogeneity (I2>89%) was considerably reduced in females to 4.4% and 30.1% for PChE and EChE, respectively. Funnel plots indicated no publication bias. However, Egger's regression confirmed the symmetry of the data points for PChE and WBChE activities with a significant effect on EChE. This meta-analysis showed normal reference values of PChE, EChE, and WBChE activities, measured by a modified electrometric method, in healthy adult humans.

## INTRODUCTION

Pesticides of organophosphorus and carbamate compounds are extensively used to control insects in public health, agriculture, and veterinary clinical practice [1–3]. These insecticides exert various manifestations of toxicity ranging from acute to chronic forms in man and animals [3–7]. The basic biochemical mechanism of the toxic action of organophosphates is irreversible inhibition of cholinesterase (ChE) activity in nervous tissues, whereas that of carbamates is reversible [4–6]. The result of such an enzyme inhibition is the production of a toxidrome of nicotinic, muscarinic, and central nervous system effects [4, 5]. Therefore, measurements of blood ChE activities, namely those of erythrocytes (EChE; true ChE; EC 3.1.1.7), plasma (PChE), or serum (pseudo or butyryl ChE; EC 3.1.1.8) and whole blood (WBChE) of man and animals are used to ascertain exposure to antiChE pesticides in suspected cases of poisoning and to interpret the extent of their health hazards [8–15]. In addition, blood ChE testing is used periodically to evaluate the health status of pesticide handlers or applicators [11, 13–15]. Generally, a decrease in PChE and/or EChE activity by 20 to 30% from baseline values suggest exposure to antiChE pesticides, whereas increased inhibition of the enzyme activity up to 50% confirms the diagnosis of antiChE poisoning [11, 13, 15–17]. Hospitalization might be needed when the inhibition of ChE activity exceeds 50% [4–7, 13]. Certain pre-exposure and post-exposure plans, such as the California plan, exist to assess the baseline blood ChE activity to overcome individual variances in enzyme activity and to aid in the diagnosis of antiChE poisoning cases in people with pre-exposure ChE activities on record in order to prevent overexposure to pesticides [10, 11, 13, 14]. In the absence of pre-exposure blood ChE activities on record, normal reference ChE values of healthy subjects are necessary for clinical assessment of pesticide poisoning [4, 5, 9, 11, 13–17].

Several laboratory and field methods are available to determine blood ChE activities. These include, but are not limited to, spectrophotometric, electrometric (potentiometric), radiometric, biosensors, and manometric procedures [18–25]. The electrometric methods are simple, accurate, and do not require elaborate instrumentations for ChE determination as other methods [18, 21–23, 25]. The original electrometric method of Michel [26] has been widely used to assay blood ChE activities in man with limited applications in animals because of the inherent variations of ChE activities in their blood components [18, 22, 23, 25]. Furthermore, the Michel electrometric method requires varying phosphate buffer preparations and sample volumes and the incubation period in the water bath takes more than one hour [18, 21, 26, 27]. Another aspect of the Michel method is that it is not advocated for measuring blood ChE in cases of carbamate poisoning because of the extent of sample dilution that occurs in the procedure and the long incubation time which facilitates decarbamylation of the inhibited enzyme [18, 23, 25]. Basically, the electrometric method utilizes the decrease in the pH of the enzymatic reaction medium after the addition of the substrate (acetylcholine), which binds to the ChE and is then hydrolyzed into choline and acetic acid [23 ,25, 26]. Based on this principle, various electrometric modifications have been reported to measure ChE activity in men and animals [18, 22, 23, 27, 28]. One of these methods is a modified electrometric determination of blood ChE activity reported in men [28–30]. The method has also been used in various animal species with different incubation times of the enzymatic reaction medium for monitoring *in vivo* and *in vitro* blood ChE inhibitions induced by organophosphates and carbamate insecticides [28, 31–37].

The procedural details of the modified electrometric method have been adequately described for laboratory applications to measure blood ChE activities in humans [28–30] and animals [22, 23, 31–40]. However, normal reference values of blood ChE activities in humans are needed for this modified electrometric method, as they are necessary for the interpretation of exposure to antiChE pesticides, especially in the absence of baseline values [9, 11, 13, 15–18]. Furthermore, Wilson *et al*. [13] stressed that it is of utmost importance to establish normal range values for human blood ChE activities with 95% confidence intervals. This is especially true when individual baseline values of blood ChE activities are not available for periodic biomonitoring of human exposure to pesticides [13–15]. The modified electrometric method for ChE determination is relatively new, and it is an emerging topic with limited information on normal human ChE values [28–30]. The purpose of the present systematic review and meta-analysis was to report normal reference ChE activities in the blood (plasma, erythrocyte, and whole blood) as determined by various studies using the present modified electrometric method in healthy adult humans not exposed to pesticides at home or their routine work. In this context, however, after the literature review, we identified a few studies on human blood ChE activities without proper citation and/or description of the steps of the modified method mentioned above [41–44]. Therefore, in the present review, the steps of the procedure were further clarified for better referencing and laboratory application.

## METHODS AND DATA EXTRACTION

All procedures of the present review and meta-analysis were approved and conducted according to institutional regulations of the Review Board, which observes the principles of medical research expressed in the Declaration of Helsinki.

### Modified electrometric method for measuring human blood ChE activity

The steps for determining human blood ChE activities by the modified electrometric method [28–30] are identified in Table 1. It is characterized by a single incubation period (20 min at 37℃) and the use of a single barbital-phosphate buffer solution (pH 8.1) with an aqueous solution of acetylcholine iodide (7.1%) or acetylthiocholine iodide (7.5%) as a substrate. The unit of ChE activity is Delta (Δ) pH/20 min.

**Table 1:** Steps of determination of human blood (plasma, serum, erythrocytes, whole blood) cholinesterase (ChE) activity by a modified electrometric method*.

	Sample	Blank
**Distilled water**	3 ml	3 ml
**Blood sample**	0.2 ml	0.2 ml distilled water
Barbital-Phosphate buffer**	3 ml	3 ml
	Measure pH1	Measure pH1
**Acetylcholine iodide 7.1% or acetylthiocholine iodide 7.5%**	0.1 ml	0.1 ml
	Incubate at 37°C for 20 min	Incubate at 37°C for 20 min
	Measure pH2	Measure pH2
**ChE activity: Delta (Δ) pH/20 min**	(pH1–pH2) – Δ pH of blank	Δ pH=pH1–pH2

* – References: 28–30; ** – 1.237 g sodium barbital, 0.163 g potassium dihydrogen phosphate and 35.07 g sodium chloride/L of distilled water, with pH adjusted to 8.1.

### Search strategy and selection criteria

**Figure 1:**
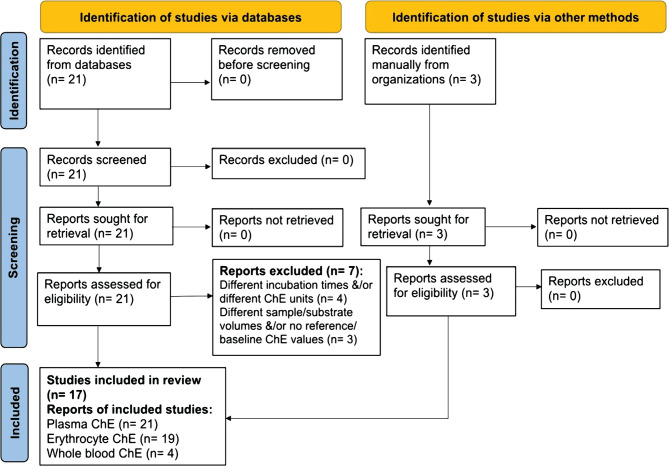
PRISMA flow diagram for systematic review of studies including searches of databases and other sources reporting reference/baseline blood cholinesterase (ChE) values in healthy adult human subjects.

The data search included studies (research articles and academic theses) that used the modified electrometric method [28, 29] for the determination of the primary outcome, which was blood (plasma, serum, erythrocytes, or whole blood) ChE activity in randomly chosen adult healthy human subjects of both sexes. The search was performed using Pubmed, Google Scholar, Scopus (Science Direct), Directory of Open Access Journals (DOAJ), and Iraqi Academic Scientific Journals (IACSJ) as of July 31, 2022. A manual search was also conducted to retrieve articles and academic theses that do not appear in the above databases. No language restriction was applied to the selection of the studies. The articles and theses were selected according to the flowchart depicted in Figure 1 for the Preferred Reporting Items for Systematic Reviews and Meta-Analysis (PRISMA) [45]. Studies using other electrometric methods for ChE determination were not included in the present meta-analysis.

All authors were involved in the selection processes, according to PRISMA. Initially, a single reviewer (FKM) screened the studies for normal values of blood ChE activities as measured by the modified electrometric method [28, 29]. The rest of the reviewers have double-screened studies to check all the records. Data extraction and calculations (mean, standard deviation, and standard error) of blood ChE activities from the published articles and theses were performed appropriately when they were not reported adequately. Values of ChE activities were extracted from the texts, tables, and figures of the studies. Subsets of data from some studies were considered different entities (reports) because they were presented as separate outcomes. Some studies needed to calculate means and SDs/SEs of ChE activity from data sets of individual baseline (or control subjects not exposed to pesticides) values. Any discrepancies were resolved through discussion among the authors of the present review.

Table 2 illustrates the inclusion/exclusion of all the studies regarding normal reference/baseline blood ChE values (measured electrometrically by the modified method) of adult human subjects not exposed to pesticides, with special notes on the excluded studies that became under scrutiny because of some technical issues. The data included for meta-analysis from the reports of human blood ChE activities were presented as mean±SD and SE (Table 3). All results of the included studies were compatible since the ChE activity in the blood component was determined by the modified electrometric method [28, 29].

**Table 2:** Inclusions of studies reporting normal (reference/baseline) blood cholinesterase activities determined by the modified electrometric method in healthy adult subjects.

Authors	Sex	PChE	EChE	WBChE	Data extraction method/exclusion	Inclusion	Reference No.
**Ahmed and Mohammad, 2004**	MF	+	+		Directly from data	Included	28
**Mahmood, 2005**	M	+	+		Calculated from baseline/control values of different experiments	Included	46
**Mohammad *et al*., 2007**	MF	+	+	+	Directly from data	Included	29
**Brifkani, 2009**	M	+	+		Directly from data	Included	47
**Garmavy, 2009**	M	+	+		Directly from data	Included	48
**Jamshidzade *et al*., 2009**	MF	+			Calculated from figure	Included	41
**Mahmood and Ahmed, 2011**	MF	+	+		Calculated from baseline values in tables	Included	49
**Mahmood, 2011**	MF	+	+		Calculated from baseline values in tables	Included	50
**Mustafa *et al*., 2011**	MF	+	+		Calculated from control values in tables	Included	51
**Al-Haseni and Yahya, 2012**	M	+			Directly from data	Included	52
**Bakand *et al*., 2012**	M	+	+		Directly from data	Included	43
**Ahmed, 2013**	M			+	Calculated from control/baseline values in tables	Included	53
**Mahmood *et al*., 2014**	MF	+	+		Calculated from control values in tables	Included	54
**Ajilore *et al*., 2017**	Not given	+	+		Calculated from baseline values in figures	Included	42
**Mahmood *et al*., 2020**	MF				Directly from data	Included	55
**Mahmood *et al*., 2021**	MF				Directly from data	Included	56
**Mohammed and Mohammad, 2022a**	M	+	+		Directly from data	Included	57
**Matti *et al*., 2006**	MF	+ serum			Directly from data	Excluded: using longer incubation time for serum ChE	58
**Ahmed and Mohammad, 2007**	MF	+	+		Calculated from data	Excluded: used same reference values of Ahmed and Mohammad, 2004	59
**Mustafa *et al*., 2008**	MF	+	+		Directly from data	Excluded: using 2 incubation times and different unit for ChE activity	60
**Janger and Al-Safar, 2009**	M	+ serum			Directly from data	Excluded: using longer incubation time and different unit for serum ChE activity	61
**Atto *et al*., 2011**	Not given	+	+		Directly from data	Excluded: using 0.1 ml sample instead of 0.2 ml in ChE assay	62
**Mostafa, 2017**	MF		+		Directly from data	Excluded: different sample and substrate volumes used; reference/baseline values not given	44
**Mustafa and Muna, 2019**	F (pregnant)	+ serum			Directly from data	Excluded: using longer incubation time and different unit for serum ChE	63

PChE – plasma cholinesterase; EChE – erythrocyte cholinesterase; WBChE – whole blood cholinesterase; M – male; F – female; MF – both sexes were used without specification; not given – gender not specified.

**Table 3:** Reports of cholinesterase activities (Δ pH/20 min) in the plasma (PChE), erythrocytes (EChE) and whole blood (WBChE) in healthy adult subjects as determined by the modified electrometric method.

Report code	Reference	Sex	n	Mean	SD	SE
**PChE**						
A	Ahmed and Mohammad, 2004	M	40	1.05	0.153	0.024
B		F	41	0.91	0.185	0.029
C	Mahmood, 2005	M	39	1.11	0.173	0.028
D	Mohammad *et al*., 2007	M	72	0.98	0.242	0.029
E		F	31	0.85	0.282	0.051
F		M	6	0.95	0.085	0.035
G	Brifkani, 2009	M	5	1.15	0.119	0.053
H	Garmavy, 2009	M	115	0.95	0.149	0.014
I		M	20	1.01	0.164	0.037
J		M	125	0.96	0.153	0.014
K	Jamshidzade *et al*., 2009	MF	10	1.0	0.615	0.194
L	Mahmood and Ahmed, 2011	MF	6	1.19	0.045	0.018
M	Mahmood, 2011	MF	3	1.15	0.021	0.012
N	Mustafa *et al*., 2011	MF	4	1.33	0.217	0.109
O	Al-Haseni and Yahya, 2012	M	50	0.93	0.283	0.040
P	Bakand *et al*., 2012	M	40	0.83	0.079	0.013
Q	Mahmood *et al*., 2014	MF	3	1.07	0.016	0.009
R	Ajilore *et al*., 2017	Not given	5	1.2	0.018	0.008
S	Mahmood *et al*., 2020	MF	30	1.34	0.383	0.07
T	Mahmood *et al*., 2021	MF	30	1.32	0.383	0.07
U	Mohammed and Mohammad, 2022a	M	15	1.49	0.155	0.040
**EChE**						
A	Ahmed and Mohammad, 2004	M	45	1.18	0.127	0.019
B		F	41	1.19	0.149	0.023
C	Mahmood, 2005	M	39	1.37	0.176	0.028
D	Mohammad *et al*., 2007	M	72	1.39	0.247	0.029
E		F	31	1.22	0.309	0.010
F		M	6	1.09	0.156	0.064
G	Brifkani, 2009	M	5	0.83	0.069	0.031
H	Garmavy, 2009	M	115	1.05	0.217	0.002
I		M	20	1.07	0.224	0.050
J		M	125	1.06	0.219	0.002
K	Mahmmod and Ahmed, 2011	MF	6	0.94	0.083	0.034
L	Mahmmod, 2011	MF	3	0.74	0.200	0.067
M	Mustafa *et al*., 2011	MF	4	1.0	0.001	0.0005
N	Bakand *et al*., 2012	M	40	0.84	0.19	0.030
O	Mahmood *et al*., 2014	MF	3	1.16	0.014	0.008
P	Ajilore *et al*., 2017	Not given	5	1.0	0.026	0.012
Q	Mahmood *et al*., 2020	MF	30	1.01	0.329	0.06
R	Mahmood *et al*., 2021	MF	30	0.97	0.657	0.12
S	Mohammed and Mohammad, 2022a	M	15	1.08	0.039	0.010
**WBChE**						
A	Mohammad *et al*., 2007	M	72	1.41	0.172	0.020
B		F	31	1.23	0.252	0.045
C	Ahmed, 2013	M	12	1.44	0.14	0.040
D		M	6	1.23	0.105	0.043

M – male; F – female; MF – both sexes were used without specification; not given – gender not specified; n – number of subjects; SD – standard deviation; SE – standard error.

### Statistical analysis

A single group meta-analysis (according to PRISMA guidelines) was conducted to evaluate the mean PChE, EChE, and WBChE activities of healthy adult subjects, using random effects model to account for the anticipated sources of heterogeneity among different studies [13, 17, 18, 21, 25]. The unit of enzyme activity reported was Δ pH/20 min [28–30]. The software programs used for the meta-analysis of PChE, EChE, and WBChE activities (Table 3) were: Open-Meta Analyst (http://www.cebm.brown.edu/openmeta/) for the forest plot to calculate effect size and weighted means with their 95% confidence intervals as well as leave-one-group assessment [64] and Meta-Essentials Version 1.5 (https://www.erim.eur.nl/research-support/meta-essentials/download/) for the funnel plot and subgroup analysis [65].

### Heterogeneity analysis

The Cochrane Q-test and I^2^ were applied to assess statistical heterogeneity and inconsistency in the reported ChE values among the reports, respectively. The level of statistical significance for the heterogeneity test (Cochrane Q-test) was set at a p-value of <0.10. After that, the forest plot was used to summarize the findings [66–69]. The minimum sample size within a report in the present meta-analysis was 3. Furthermore, funnel plot (effect size measure against standard error of ChE activity as obtained from the reports) and Egger's test were used to evaluate statistically the presence of any publication bias [68, 69]. The trim-and-fill analysis was also included for the adjustment of potentially missing studies.

## RESULTS

Figure 1 depicts the PRISMA flow chart for selecting studies that included normal reference/baseline blood ChE activities using the modified electrometric method [28, 29]. After applying the exclusion criteria (Table 2), the initial list of selected studies (24) was narrowed down to 17 (14 articles and 3 theses), which comprised 21, 19, and 4 separate reports of normal reference/baseline PChE, EChE and WBChE activities, respectively, in healthy adult males/and or females (Figure 1, Table 2). These articles and theses were published between 2004 and 2022, with sample sizes of 690, 635, and 121 subjects for PChE, EChE, and WBChE activities, respectively (Table 3). They represented normal (or baseline) blood ChE activities in adult males and/or females recruited for ChE studies as apparently healthy subjects from several regions, including Iraq, Iran, Egypt, and Nigeria (Table 2).

### Meta-analysis

**Figure 2:**
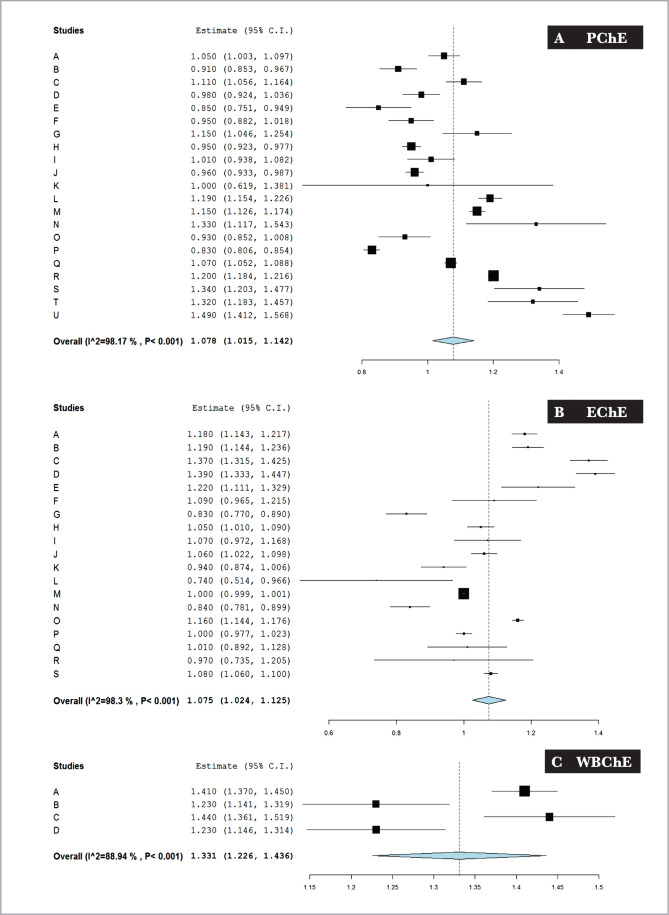
The forest plot and cumulative meta-analysis of cholinesterase activities (Δ pH/20 min) in the plasma (A – PChE), erythrocytes (B – EChE) and whole blood (C – WBChE) reported in healthy adult subjects by various studies using the modified electrometric method.

Using the randomized effects model, single group meta-analysis of the present reports revealed that the normal reference values of the means (effect sizes in the forest plots) with 95% CI of PChE, EChE, and WBChE activities of adult healthy subjects were 1.078 (1.015, 1.142), 1.075 (1.024, 1.125) and 1.331 (1.226, 1.436), respectively (Figure 2 A–C). The percentages of weights of individual reports obtained from the forest plots are shown in Table 4. The % weights of the reports for the three blood ChE activities were close to each other within each ChE measurement. The minimum and maximum weights were 1.81–5.29%, 2.61–6.12%, and 23.75–27.48% for the PChE, EChE, and WBChE activities, respectively (Table 4). Table 5 summarizes the findings of the meta-analysis of blood ChE activities.

**Table 4:** Percentages of weights of the reports of cholinesterase activities in the plasma (PChE), erythrocytes (EChE) and whole blood (WBChE) following the forest plot*.

Report code	PChE	EChE	WBChE
**A**	5.154%	5.918%	27.475%
**B**	5.091%	5.821%	23.753%
**C**	5.108%	5.693%	24.598%
**D**	5.096%	5.667%	24.175%
**E**	4.691%	4.748%	
**F**	5.000%	4.433%	
**G**	4.635%	5.616%	
**H**	5.256%	5.891%	
**I**	4.966%	4.953%	
**J**	5.258%	5.904%	
**K**	1.806%	5.523%	
**L**	5.218%	2.721%	
**M**	5.269%	6.116%	
**N**	3.310%	5.640%	
**O**	4.906%	6.079%	
**P**	5.266%	6.040%	
**Q**	5.285%	4.572%	
**R**	5.291%	2.606%	
**S**	4.244%	6.059%	
**T**	4.244%		
**U**	4.906%		

* – The data were obtained from the forest plots determined by the Open-Meta.

**Table 5:** Summary results of random-effects model meta-analysis of cholinesterase activities (Δ pH/20 min) in the plasma (PChE), erythrocytes (EChE) and whole blood (WBChE) of healthy adult humans.

Variable	PChE	EChE	WBChE
**Forest plot**			
Effect size	1.078	1.075	1.331
95% confidence interval	1.015, 1.142	1.024, 1.125	1.226, 1.436
Standard error	0.032	0.026	0.054
p-value	<0.001	<0.001	<0.001
Minimum report weight	1.81%	2.61%	23.75%
Maximum report weight	5.29%	6.12%	27.48%
**Heterogeneity**			
tau^2^	0.019	0.011	0.010
Q	1090.5 (df=20)	1059.9 (df=18)	27.124 (df=3)
Het. p-value	<0.001	<0.001	<0.001
I^2^	98.2%	98.3%	88.9%
**Subgroup analysis**			
**Females**			
tau^2^	0.00	0.00	Only one report
Q	1.05	1.43	
Het. p-value	0.306	0.232	
I^2^	4.4%	30.1%	
**Males**			
tau^2^	0.02	0.00	0.01
Q	334.6	424.8	16.4
Het. p-value	<0.001	<0.001	<0.001
I^2^	97%	97.9%	87.8%
**Males and females**			
tau^2^	0.00	0.01	Not available
Q	74	416.8	
Het. p-value	<0.001	<0.001	
I^2^	91.9%	98.8%	
**Leave-one-out analysis**			
Minimum leave-one-out value	1.057	1.056	1.294
Maximum leave-one-out value	1.090	1.089	1.364
**Funnel plot, Egger test**			
t-test	-0.37	2.43	-1.36
p-value	0.72	0.026	0.308
No. of imputed points	3	1	0

### Heterogeneity analysis

The I^2^ statistical test was used to examine the variations among the studies as a result of heterogeneity; the value of I^2^ could range from 0% (no heterogeneity) to 100% (high heterogeneity) [66–68]. The heterogeneity values of the PChE, EChE, and WBChE activities were 98.2, 98.3, and 88.9%, respectively, with statistically significant Cochrane Q-test values (p<0.0001), indicating high levels of heterogeneity (Table 5).

### Subgroup analysis

Because of the considerable heterogeneity (>88%) in the meta-analysis (Table 5), we conducted a subgroup analysis on the available data for males (M), females (F), or both (MF) when blood samples were used without discrimination to identify the possible source of heterogeneity among the reports of blood ChE activities. Subgroup analysis indicated that the heterogeneity (%) was considerably reduced for the PChE and EChE activities in females (consisting of two reports) to I^2^ values of 4.4% and 30.1%, respectively (Table 5). The I^2^ values of males and both sexes (MF) used indiscriminately remained significantly high (Table 5). The evidence for WBChE subgroup analysis was not conclusive because of the limitation of the available reports (1 F and 3 M) (Table 3).

### Sensitivity (leave-one-group) analysis

**Figure 3:**
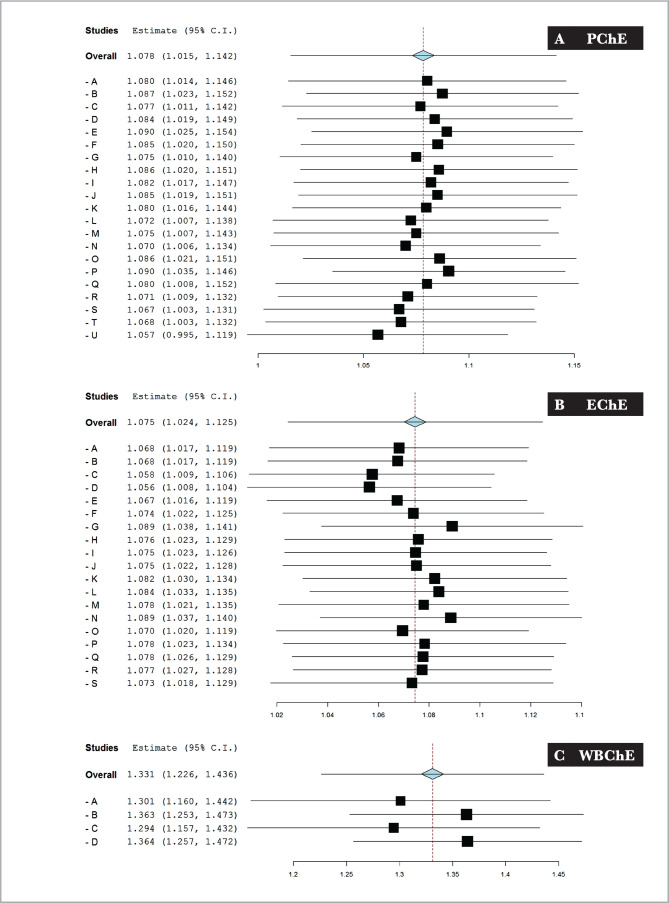
The forest plot of one-report leave-out sensitivity analysis of cholinesterase activities (Δ pH/20 min) in the plasma (A – PChE), erythrocytes (B – EChE) and whole blood (C – WBChE) reported in healthy adult subjects by various studies using the modified electrometric method.

Leave-one-group meta-analysis was conducted to assess the sensitivity of the analysis and stability of the effect size estimates. According to the resultant forest plots of PChE, EChE, and WBChE activities, it was revealed that the percentages of changes in the effects sizes ranged between 1 to 2% for both PChE and EChE activities and between 2.5 to 3% for the WBChE activity (Figure 3 A–C, Table 5). The overall result of the leave-one-group analysis did not significantly affect the effect size outcome.

### Publication bias (funnel plot)

**Figure 4:**
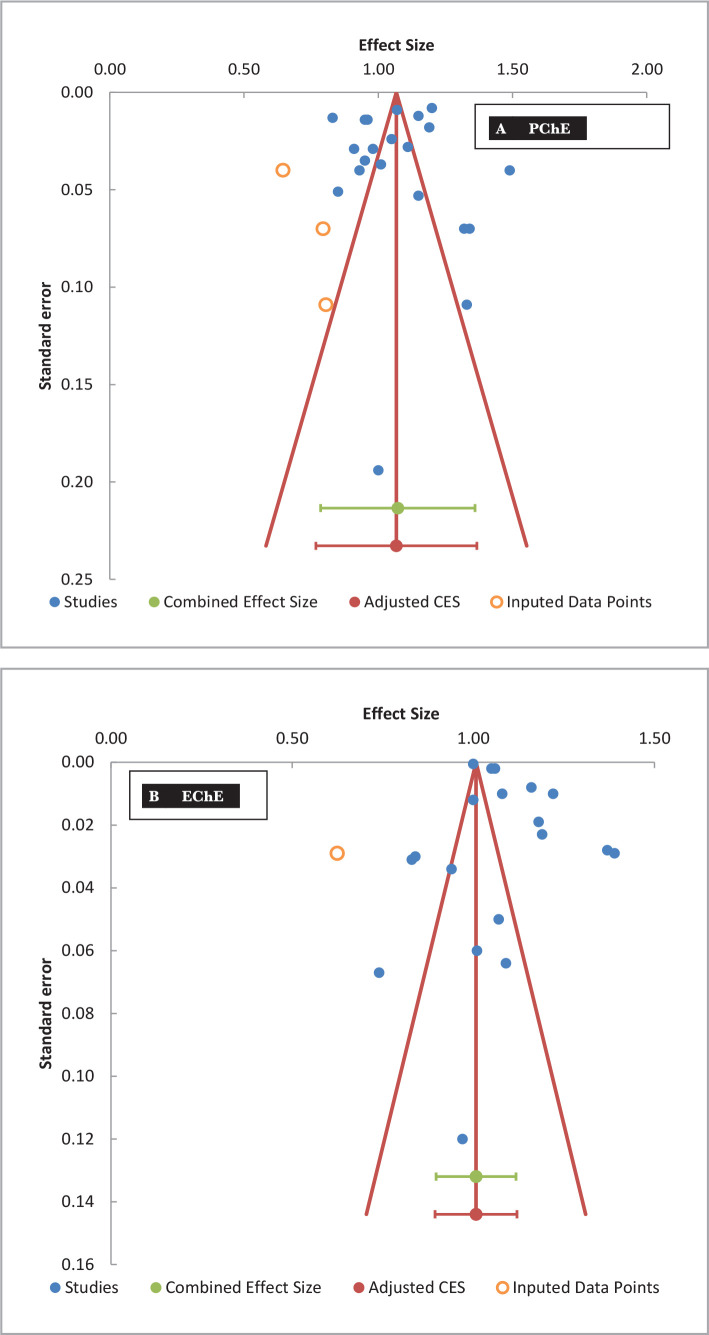

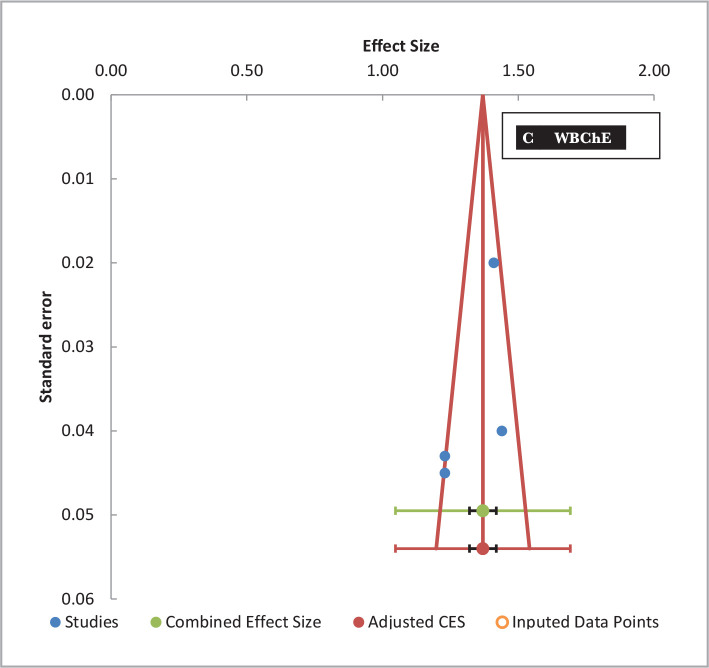
The funnel plot (publication bias) cholinesterase activities (Δ pH/20 min) in the plasma (A – PChE), erythrocytes (B – EChE) and whole blood (C – WBChE) in healthy adult subjects reported by various studies using the modified electrometric method. X-axis represents the mean cholinesterase activity (effect size) and the Y-axis represents the standard error of the mean. The trim-and-fill analysis was also included by the imputed data points (open circles) for the adjustment of potentially missing studies in PChE and EChE.

The funnel plot and Egger's test were used to assess the possibility of publication bias. The standard errors (y-axis) were plotted against the effect sizes (x-axis). The asymmetrical distribution of the data points of the reports suggested the presence of such a condition. However, visual examination of the funnel plots of PChE, EChE, and WBChE activities indicated no publication bias (Figure 4 A–C). Further analysis with Egger's weighted regression confirmed the symmetry of the data points for PChE and WBChE activities with only a significant bias for that of the EChE (Table 5). The trim-and-fill analysis (adjustment for the potentially missing studies) of the funnel plot for the EChE activity revealed only one imputed point to adjust the combined effect size (Figure 4 B). However, all three ChE activities maintained their combined effect sizes after the trim-and-fill analysis for adjustment (Figure 4 A–C).

## DISCUSSION

Measuring blood (plasma, serum, erythrocyte, or whole blood) ChE activities is an important biomonitoring tool to assess exposure to organophosphate and carbamate pesticides, especially among agriculture workers, [1, 10, 13, 15–17, 43, 57]. The present modified electrometric method is a suitable assay tool, especially in laboratories with limited resources [28–30]. We would recommend it because of the simplicity of the procedure and its applicability to agriculture workers [43, 57, 59]. Furthermore, the modified electrometric method can be used in various animal species with minor variations in the incubation time of the enzymatic reaction medium since animals can be monitored for exposure to antiChE pesticides therapeutically, environmentally, or experimentally [22, 23, 27, 28, 31–34, 70, 71]. The method can also be used for *in vitro* evaluation of antiChE drugs and pesticides [28, 29, 31–37, 39, 71].

The present meta-analysis reports the reference ChE activities in the plasma, erythrocytes, and whole blood of healthy adult humans. These values could form the basis for comparison following exposure of workers to antiChE pesticides, especially in the absence of pre-exposure values [9, 11, 13, 15–18]. However, the results of the present meta-analysis should not be taken as final or for granted since there could be sources of ChE variations due to age, gender, and demographic characteristics of the subjects [7–11, 13–19, 21, 25, 27]. Therefore, an updated continuous meta-analysis on normal ChE activities determined by the modified electrometric method is further warranted.

In the present meta-analysis, subgroup analysis indicated that the heterogeneity (%) was considerably reduced in the PChE and EChE activities of females, which consisted of only two reports. The I^2^ values of males and both sexes (MF) remained significantly high, with no conclusive evidence for WBChE subgroup analysis because of the limited available reports (Tables 2, 3 and 5). Based on these findings and the fact that about half of the studies used both males and females indiscriminately (Table 2), the gender effect (with limited reports in males and females) on blood ChE could not be ascertained at present. Furthermore, gender differences in blood ChE activities were not found previously [29]. However, other sources of ChE variations should not be omitted in interpreting blood ChE activities of pesticide-exposed subjects because of the subjects' physiological conditions and demographic characteristics [7–11, 13–19, 21, 25, 27].

We noticed some studies misquoted the modified electrometric method or missed some of the important details of the procedure, such as the incubation time and concentration of the substrate [41–44]. However, Table 1 and a recent communication [30] clarify these issues.

In the present review, we report normal reference WBChE values in humans with no heterogeneity (Figure 2 C). This is based on two references only [29, 53], and being a limited scale finding, further studies are needed to better establish normal reference WBChE values determined by the present electrometric method. Measuring WBChE activities in humans is especially useful under field conditions [8, 9, 13, 15, 18, 21, 25].

## CONCLUSION

This study and the meta-analysis showed normal reference values of PChE, EChE, and WBChE activities, measured by a modified electrometric method, in adult healthy humans. These blood ChE values could form the basis for interpreting workers' exposure to antiChE pesticides. Further studies are needed to update meta-analysis on normal blood ChE activities determined by the modified electrometric method and on the sources of blood ChE variations due to age, gender, and demographic characteristics of the subjects.
